# Grafting with rootstocks promotes phenolic compound accumulation in grape berry skin during development based on integrative multi-omics analysis

**DOI:** 10.1093/hr/uhac055

**Published:** 2022-03-14

**Authors:** Fuchun Zhang, Haixia Zhong, Xiaoming Zhou, Mingqi Pan, Juan Xu, Mingbo Liu, Min Wang, Guotian Liu, Tengfei Xu, Yuejin Wang, Xinyu Wu, Yan Xu

**Affiliations:** State Key Laboratory of Crop Stress Biology in Arid Areas, College of Horticulture, Northwest A&F University, 712100, Yangling, Shaanxi, China; Institute of Horticulture Crops, Xinjiang Academy of Agricultural Sciences (Key Laboratory of Genome Research and Genetic Improvement of Xinjiang Characteristic Fruits and Vegetables), 830091, Urumqi, Xinjiang, China; Institute of Horticulture Crops, Xinjiang Academy of Agricultural Sciences (Key Laboratory of Genome Research and Genetic Improvement of Xinjiang Characteristic Fruits and Vegetables), 830091, Urumqi, Xinjiang, China; Institute of Horticulture Crops, Xinjiang Academy of Agricultural Sciences (Key Laboratory of Genome Research and Genetic Improvement of Xinjiang Characteristic Fruits and Vegetables), 830091, Urumqi, Xinjiang, China; Institute of Horticulture Crops, Xinjiang Academy of Agricultural Sciences (Key Laboratory of Genome Research and Genetic Improvement of Xinjiang Characteristic Fruits and Vegetables), 830091, Urumqi, Xinjiang, China; Institute of Horticulture Crops, Xinjiang Academy of Agricultural Sciences (Key Laboratory of Genome Research and Genetic Improvement of Xinjiang Characteristic Fruits and Vegetables), 830091, Urumqi, Xinjiang, China; State Key Laboratory of Crop Stress Biology in Arid Areas, College of Horticulture, Northwest A&F University, 712100, Yangling, Shaanxi, China; Institute of Horticulture Crops, Xinjiang Academy of Agricultural Sciences (Key Laboratory of Genome Research and Genetic Improvement of Xinjiang Characteristic Fruits and Vegetables), 830091, Urumqi, Xinjiang, China; Institute of Horticulture Crops, Xinjiang Academy of Agricultural Sciences (Key Laboratory of Genome Research and Genetic Improvement of Xinjiang Characteristic Fruits and Vegetables), 830091, Urumqi, Xinjiang, China; State Key Laboratory of Crop Stress Biology in Arid Areas, College of Horticulture, Northwest A&F University, 712100, Yangling, Shaanxi, China; State Key Laboratory of Crop Stress Biology in Arid Areas, College of Horticulture, Northwest A&F University, 712100, Yangling, Shaanxi, China; State Key Laboratory of Crop Stress Biology in Arid Areas, College of Horticulture, Northwest A&F University, 712100, Yangling, Shaanxi, China; Institute of Horticulture Crops, Xinjiang Academy of Agricultural Sciences (Key Laboratory of Genome Research and Genetic Improvement of Xinjiang Characteristic Fruits and Vegetables), 830091, Urumqi, Xinjiang, China; State Key Laboratory of Crop Stress Biology in Arid Areas, College of Horticulture, Northwest A&F University, 712100, Yangling, Shaanxi, China

## Abstract

In viticulture, grafting has been practiced widely and influences grape development as well as berry and wine quality. However, there is limited understanding of the effects of rootstocks on grape phenolic compounds, which are located primarily in the berry skin and contribute to certain sensory attributes of wine. In this study, scion–rootstock interactions were investigated at the green-berry stage and the veraison stage when grapevines were hetero-grafted with three commonly used rootstock genotypes (5BB, 101-14MG, and SO4). Physiological investigations showed that hetero-grafts, especially CS/5BB, contained higher concentrations of total proanthocyanidins (PAs) and various PA components in berry skins compared with the auto-grafted grapevines. Further metabolomics analysis identified 105 differentially accumulated flavonoid compounds, the majority of which, including anthocyanins, PAs, and flavonols, were significantly increased in the berry skins of hetero-grafted grapevines compared with auto-grafted controls. In addition, transcriptomic analysis of the same samples identified several thousand differentially expressed genes between hetero-grafted and auto-grafted vines. The three rootstocks not only increased the transcript levels of stilbene, anthocyanin, PA, and flavonol synthesis genes but also affected the expression of numerous transcription factor genes. Taken together, our results suggest that hetero-grafting can promote phenolic compound accumulation in grape berry skin during development. These findings provide new insights for improving the application value of grafting by enhancing the accumulation of nutritious phenolic components in grape.

## Introduction

Grapevine (*Vitis vinifera* L.) is an economically important fruit crop that is well adapted to growth in a wide range of climatic conditions and is thus cultivated worldwide [1, 2]. Grapes have significant economic value, as they are not only consumed fresh but also used for juice and wine making. In addition, grapes are rich in nutrients with health benefits to humans [3]. In China, the scale of grape cultivation has increased rapidly over the past few decades. Because of its unique geographic location, light, and rich variety of resources, Xinjiang has become the largest grape producing area in China. The grapevine cultivation area has reached 26 000 hectares, and the Xinjiang government plans to develop the grape industry on a larger scale in the next decade. However, the quality of grapes in Xinjiang generally suffers from the problem of low contents of phenolic compounds such as proanthocyanidins (PAs).

Grape berry skin is enriched in phenolic compounds, which include non-flavonoid compounds such as stilbenes, as well as flavonoid compounds such as flavonols, PAs, and anthocyanins [4, 5]. These phenolic compounds have multiple biological functions; they provide protection against biotic and abiotic stresses, contribute to the taste and astringency of wine, and also act as potential dietary antioxidants with health benefits to humans [6]. In grapevine, anthocyanins are responsible for red pigmentation and accumulate mainly during ripening in berry skins [7]. Flavonols are responsible for protection against UV radiation and are synthesized in berry skins during the early stages of berry development and during ripening [8]. PAs are polymers of flavan-3-ol units such as catechin, epicatechin, and epigallocatechin, and they contribute to the taste and astringency of wine [9]. PAs are synthesized in the early stages of berry development, and their synthesis is completed around veraison. These phenolic compounds are synthesized via the flavonoid pathway, which has been well characterized. Accumulation of phenolic compounds in grape skins is a complex physiological and biochemical process, and many factors, including grape variety, yield, and exogenous stimuli, influence their accumulation [10, 11]. It has been reported that a series of transcription factors such as R2R3-MYB, WRKY, and bHLH proteins are involved in the transcriptional regulation of flavonoid biosynthetic pathway genes [12–14]. In recent years, some studies have revealed that rootstock genotype has a possible influence on the accumulation of some phenolic compounds [15–17]. Our lab previously found that rootstock genotypes such as 5BB and 101-14MG affect tannin accumulation in the Cabernet Sauvignon variety. Therefore, studying the mechanisms by which rootstocks influence PAs has both basic and applied significance for improving the problem of low PA content in Xinjiang grapes.

In the late 19th century, grape phylloxera (*Daktulosphaira vitifoliae* Fitch) spread in Europe and caused serious damage to vineyards worldwide [18]. Later on, phylloxera-resistant rootstocks derived from American *Vitis* species were used successfully and represent the longest use of a biological control strategy [19]. Although the grape cultivation area in China continues to expand, most vines are own-rooted cultivars that are not resistant to pathogens and pests, seriously restricting the development of the grape industry. Grafted grape cultivation in China began only in the 1960s. Grape grafting is a widely used technique with many advantages. Rootstocks are not only used to treat various diseases and pests but are also tolerant to diverse abiotic stress conditions such as salinity, drought, and alkalinity [20, 21]. In addition, rootstocks can improve the internal and external quality of scion varieties, regulate grape maturity, and increase yield [22]. The rootstock, as a nutrient transport channel of the grapevine, also plays a very important role in mineral element metabolism and can therefore cause the vine to show differences in growth or significant changes in fruit quality and yield. Some rootstocks can advance or delay the grape harvest time. However, studies of the molecular processes that govern scion–rootstock interactions are still scarce. Therefore, studying the effects of rootstocks on grape growth and PA accumulation can provide an important basis for improving PAs and other phenolic compounds in Xinjiang grapes.

In recent years, a number of -omics techniques such as transcriptomics, proteomics, and metabolomics have been used to study rootstock-mediated effects involved in the modification of gene expression and secondary metabolites in grape. An earlier microarray study on the berry skin of Norton grape revealed that transcript levels of *PR-1* and stilbene synthase genes clearly increased, which may have contributed to developmentally regulated resistance [23]. In another study, the analysis of metabolome data showed that the Shiraz cultivar had higher levels of organic acids, sugars, and phenolic compound precursors than the Cabernet Sauvignon cultivar. Accordingly, transcript profiling showed that Shiraz had a large number of upregulated genes related to the entire polyphenol pathway and to stress processes [24]. Furthermore, a study combining transcriptomics and metabolomics on leaves of the Gaglioppo variety showed that levels of metabolites and genes involved in defense responses differed among diverse rootstocks [25]. However, limited data are available regarding the effects of different rootstocks on dynamic changes in the metabolome and transcriptome in grape berry skins during different developmental stages.

This study investigated how different scion–rootstock combinations influence physiological parameters, gene expression, and metabolites in grape berry skin in order to determine the actual effects of rootstocks on phenolic compound accumulation during berry development. The climate in Xinjiang is arid, and the soil salinity is high. 5BB and SO4 rootstocks have been reported to confer tolerance to drought and salinity, whereas 101-14MG can promote grape fruit coloring; all are currently used for grapevine grafting worldwide. The selection of these three rootstocks meets the needs of grape-producing areas in Xinjiang. Therefore, this study was set up using the Cabernet Sauvignon variety grafted onto these three commonly used rootstock genotypes and also auto-grafted onto itself. Sugars, organic acids, and PA content were measured at the green-berry stage and the veraison stage. Then variation in flavonoid accumulation was evaluated using a metabolomics approach. Together with the metabolic analysis, gene expression changes in the berry skin affected by rootstocks were analyzed using a transcriptomics approach.

## Results

### Rootstocks promote the accumulation of sugars and organic acids during grape berry development

**Figure 1:**
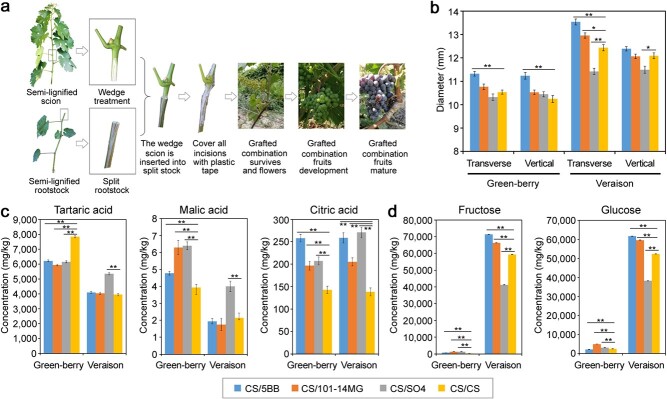
Effects of rootstocks on physiological indices of grape berries. **a** Grafting process used in this study. **b** Effects of rootstocks on grape berry size at two developmental stages. **c** Effects of rootstocks on the organic acid concentrations of grape berries. **d** Effects of rootstocks on fructose and glucose concentrations of grape berries. “^*^” indicates a statistically significant difference at *p* < 0.05 between the hetero-graft (CS/5BB, CS/101-14MG, or CS/SO4) and the auto-grafted control, and “^**^” indicates a statistically significant difference at *p* < 0.01 between the hetero-graft and the auto-grafted control.

*V. vinifera* cv. Cabernet Sauvignon (CS), a widely planted variety of red grape, was used in this study. CS was hetero-grafted with three commonly used rootstock genotypes (5BB, 101-14MG, and SO4) and auto-grafted with itself (Fig. 1a). The grape berries of each scion–rootstock combination were harvested at the green-berry stage (45 days after flowering) and the veraison stage (75 days after flowering) (Fig. S1). At the green-berry stage, the berry size of the CS/5BB hetero-graft was significantly larger than those of CS/101-14MG, CS/SO4, and the auto-grafted control (Fig. 1b). The berry skin color shifted from green to purple at the veraison stage, and the berry size of CS/5BB was still the largest. CS/SO4 had a slightly smaller berry size than the auto-grafted control (Fig. 1b). These results showed that the 5BB rootstock promoted the development of grape berries, whereas SO4 had the opposite effect.

Sugar and acid contents determine the organoleptic quality and flavor of the grape [26]. We therefore detected the sugar and organic acid contents of grape berries to investigate the influence of the rootstocks on grape quality. Initially, we detected the contents of malic acid, tartaric acid, and citric acid. As shown in Fig. 1c, the concentrations of two important organic acids, tartaric acid and malic acid, were much higher at the green-berry stage than at the veraison stage, consistent with the conventional phenomenon in which organic acids are synthesized and reach maximal concentrations during initial berry growth (Phase I) [27]. At the green-berry stage, the amounts of citric acid and malic acid were much higher in the hetero-grafts with the three rootstocks than in the auto-grafted control, whereas tartaric acid levels were much lower in the hetero-grafts (Fig. 1c). Intriguingly, the rootstock genotype SO4 exhibited higher concentrations of malic acid and tartaric acid than the auto-grafted control, whereas 5BB and 101-14MG had little influence at the veraison stage. All three rootstocks significantly increased the content of citric acid compared with the auto-grafted control at the veraison stage (Fig. 1c). Sugar accumulation begins during phase II, which is characterized as a lag phase [27]. Consistent with this phenomenon, the concentrations of fructose and glucose were relatively low at the green-berry stage and increased markedly at the veraison stage (Fig. 1d). In contrast to the auto-grafted control, 5BB and 101-14MG had much higher contents of fructose and glucose at the veraison stage, whereas SO4 showed the opposite pattern. In addition, the rootstock genotype 5BB had the most abundant contents of fructose and glucose among the three tested rootstocks (Fig. 1d). These results suggested that the three rootstocks promoted the accumulation of organic acids and sugars in grape berries during berry development.

### Rootstocks increase the proanthocyanidin content of berry skin

**Figure 2:**
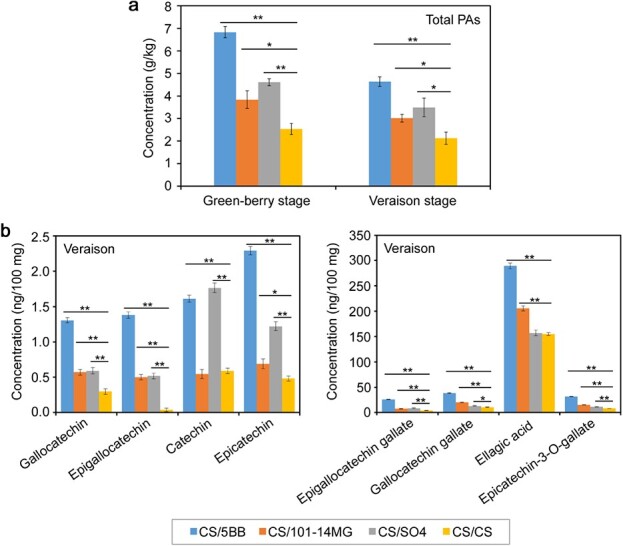
Effects of rootstocks on total PAs and PA components of berry skin. **a** Effects of rootstocks on total PA content at two developmental stages. **b** Effects of rootstocks on the concentrations of different PA components at the veraison stage. “^*^” indicates a statistically significant difference at *p* < 0.05 between the hetero-graft (CS/5BB, CS/101-14MG, or CS/SO4) and the auto-grafted control, and “^**^” indicates a statistically significant difference at *p* < 0.01 between the hetero-graft and the auto-grafted control.

Proanthocyanidins (PAs) are flavan-3-ol oligomers that play an important role in wine astringency and bitterness; they are located primarily in the berry skins and seeds [28]. To globally evaluate the effects of scion–rootstock interactions on PA content in berry skins, total PA concentration was measured. As shown in Fig. 2a, the total PA concentration was much higher in the hetero-grafts than in the auto-grafted control at both developmental stages. CS/5BB produced the highest amount of PA, followed by CS/SO4 and CS/101-14MG. Notably, the total PA content of CS/5BB was more than twice that of the auto-grafted control (Fig. 2a). The PA concentration of each scion–rootstock combination decreased at the veraison stage compared with the green-berry stage.

The most important PAs are flavan-3-ol (catechin) and its condensed forms, including gallocatechin, epicatechin, epigallocatechin, epigallocatechin gallate, gallocatechin gallate, ellagic acid, and epicatechin-3-O-gallate. We detected these PA components in berry skin at the veraison stage. Notably, seven components (epigallocatechin, gallocatechin, epigallocatechin gallate, epicatechin, gallocatechin gallate, ellagic acid, and epicatechin-3-O-gallate) exhibited significantly higher concentrations in all three hetero-grafted grapevines than in the auto-grafted control (Fig. 2b). The accumulation of catechin was much higher in CS/5BB and CS/SO4 than in the auto-grafted control, whereas CS/101-14MG showed no difference. Among the three hetero-grafted combinations, we observed that CS/5BB had the highest concentrations of seven PA components except catechin, which was most abundant in CS/SO4. Further comparisons between CS/101-14MG and CS/SO4 showed that the concentrations of gallocatechin gallate, ellagic acid, and epicatechin-3-O-gallate were higher in CS/101-14MG, whereas the remaining five PA components were higher in CS/SO4 (Fig. 2b). Taken together, these results show that the three rootstocks promoted the accumulation of PAs relative to the auto-grafted control, and CS/5BB had the most abundant PA components.

### Metabolomics reveals differential flavonoid accumulation among rootstocks

**Figure 3:**
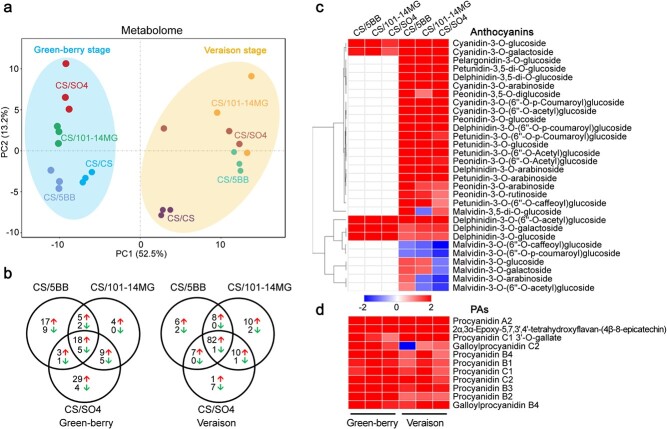
Differentially accumulated flavonoid metabolites in berry skins affected by rootstocks. **a** PCA scatterplot of berry skin samples based on metabolomics data. **b** Venn diagram of counts of differentially accumulated flavonoid metabolites among three hetero-grafts. The differentially accumulated flavonoid metabolites were identified by comparing the abundances of metabolites between hetero-grafts (CS/5BB, CS/101-14MG, or CS/SO4) and the auto-grafted control. The number on the left of the red arrow indicates the number of increased metabolites, and the number on the left of the green arrow indicates the number of decreased metabolites. **c** Cluster heatmap of different anthocyanin compounds affected by the three rootstocks. **d** Cluster heatmap of different PA compounds affected by the three rootstocks. Different colors represent an increase (red) or decrease (blue) in the metabolite fold-change as indicated in the color index. The fold-change and the abundance of each metabolite can be found in Table S1.

PA is a key compound of the flavonoids, which also include several other important compounds such as anthocyanins, flavonols, flavanols, and dihydroflavones [29]. We used a widely-targeted liquid chromatography–mass spectrometry (LC–MS) method to perform comprehensive profiling of flavonoids in grape berry skins after grafting with three different rootstocks. In total, 184 flavonoid compounds were identified in the berry skin samples, including 11 PAs, 51 flavonols, 16 flavanols, 8 chalcones, 12 dihydroflavones, 43 flavonoids, 30 anthocyanins, 2 flavonoid carbonosides, and 11 tannins (Table S1). All the identified flavonoid compounds were subjected to PCA to visualize the general trend in flavonoid changes associated with the scion–rootstock combinations and developmental stages. PCA not only showed the clear separation between the two developmental stages but also revealed that the general flavonoid composition of berry skins differed markedly between the hetero-grafts and the auto-grafted control (Fig. 3a). Comparison of hetero-grafts and the auto-grafted control showed that 105 flavonoid compounds were differentially accumulated at the green-berry stage, and 136 flavonoid compounds were differentially accumulated at the veraison stage. The contents of the majority of flavonoids were significantly increased by grafting with the three rootstocks (Fig. 3b). Intriguingly, all three rootstocks promoted the accumulation of 82 specific flavonoid compounds at the veraison stage (Fig. 3b, Table S1), and these were mainly composed of PAs and flavonols.

Anthocyanins are mainly responsible for the color palette and flavonols for UV protection and wine astringency [30]. In this study, only five anthocyanin compounds were detected at the green-berry stage, whereas 30 anthocyanin compounds were identified at the veraison stage (Fig. 3c, Table S1), consistent with the phenomenon in which grape berry skins usually began to accumulate anthocyanins at veraison [23]. There was a progressive accumulation of anthocyanins across berry development in the three hetero-grafts. At the green-berry stage, the amounts of all the five detected anthocyanin compounds were significantly higher in the hetero-grafts than in the auto-grafted control. At the veraison stage, 63% of the anthocyanin compounds exhibited higher amounts in at least two of the three scion–rootstock combinations compared with the auto-grafted control (Fig. 3c, Table S1). Notably, five anthocyanin derivatives, delphinidin-3-O-glucoside, delphinidin-3-O-galactoside, delphinidin-3-O-(6′′-O-acetyl) glucoside, petunidin-3-O-(6′′-O-acetyl) glucoside, and cyanidin-3-O-glucoside, were the most abundant anthocyanins in all the analyzed hetero-grafts (Fig. 3c, Table S1). These results indicated that the three rootstocks stimulated anthocyanin production, especially at the veraison stage.

PAs are predominantly found in the grape berry skin and are key factors that contribute to the quality of wine. We observed that PA accumulation was lower at the veraison stage than at the green-berry stage. A total of 11 PA derivatives were identified, and all of them showed high diversity in accumulation at both developmental stages (Fig. 3d, Table S1). Four PA derivatives, procyanidin B2, procyanidin B4, procyanidin A2, and 2α,3α-epoxy-5,7,3′,4′-tetrahydroxyflavan-(4β-8-epicatechin), showed higher levels in the three hetero-grafts, and the remaining seven PA derivatives were also abundant in at least one hetero-graft at the green-berry stage. Although PA contents decreased at the veraison stage, there was still a significant difference between hetero-grafted grapevines and the auto-grafted control. In contrast to the auto-grafted control, eight PA derivatives showed higher amounts in CS/101-14MG, and four PA derivatives were more abundant in CS/5BB and CS/SO4 at the veraison stage (Table S1), suggesting that the three rootstocks strongly promoted PA accumulation at both developmental stages.

**Figure 4:**
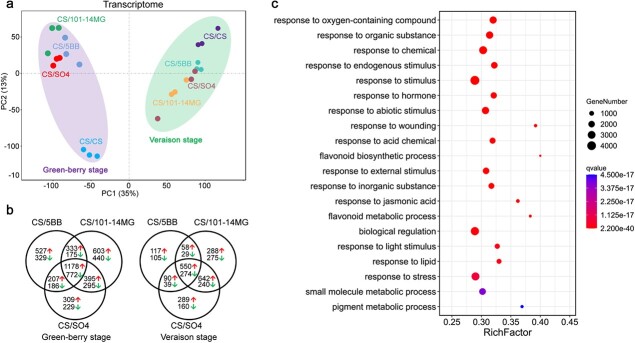
Overview of transcriptomic changes in berry skins affected by rootstocks. **a** PCA scatter plot of berry skin samples based on transcriptomic data. **b** Venn diagram of DEG numbers in different comparison groups. The DEGs were identified by comparing the expression levels of genes between hetero-grafts (CS/5BB, CS/101-14MG, or CS/SO4) and the auto-grafted control. The number on the left of the red arrow indicates the number of upregulated genes, and the number on the left of the green arrow indicates the number of downregulated genes. **c** GO enrichment of biological process (BP) terms in the comparison of the transcriptomes affected by the rootstocks.

Flavonols are a type of flavonoid with a 3-hydroxyflavone backbone that play a crucial role in the color and bitter taste of red wine by stabilizing anthocyanin pigments [4, 31]. Here, we identified 51 flavonol compounds (Table S1), which represented the most abundant flavonoids. At the two developmental stages, the majority of flavonols were differentially accumulated between hetero-grafted vines and the auto-grafted control. At the green-berry stage, CS/5BB and CS/101-14MG had lower levels of ten flavonols and increased levels of only one flavonol (Table S1). By contrast, CS/SO4 had increased levels of 21 flavonols, indicating that the three hetero-grafts exhibited differential accumulation of flavonols at this stage. Notably, 75% of the detected flavonol compounds were differentially accumulated in at least two of the three hetero-grafted combinations, and the majority were markedly increased in the three hetero-grafts at the veraison stage (Table S1). Taken together, these results show that rootstock genotypes 5BB and 101-14MG reduced flavonol levels at the green-berry stage and promoted flavonol accumulation at the veraison stage, whereas SO4 increased flavonol content at both developmental stages.

### Transcriptome overview

To investigate the rootstock effects on the grape berry skin transcriptome and to relate these changes to the observed metabolic changes, RNA-seq was carried out on the same samples used for metabolite analysis. Quality filtered reads were mapped to the *V. vinifera* genome. The majority of clean reads from each library (82.5–93.6%) were successfully aligned to the genome (Table S2), and only the uniquely aligned reads were used for the following calculations of gene expression values. On the whole, over 18 000 expressed genes were detected in each sample.

A PCA analysis was performed to evaluate sample correlation. Based on the transcriptional profiles, there was a clear separation of hetero-grafted samples and auto-grafted samples in the plot (Fig. 4a). PCA showed that data from the three hetero-grafts were more similar to each other than to data from the auto-grafted control at each developmental stage. Moreover, the distance between hetero-grafts and the auto-grafted control was much larger at the green-berry stage than at the veraison stage (Fig. 4a). The plot also clearly showed a high similarity among the three biological replicates, suggesting good reproducibility. These results indicated that the RNA-seq data were of high quality, and all data were therefore used for subsequent analysis.

### Identification and functional enrichment analysis of DEGs

Pairwise comparisons between the hetero-grafted samples (CS/5BB, CS/101-14MG, or CS/SO4) and the auto-grafted (CS/CS) sample at the same developmental stage were performed to identify differentially expressed genes (DEGs). A great many genes were found to be differentially expressed in the berry skins. A total of 3707, 4235, and 3640 DEGs were identified between hetero-grafted and auto-grafted samples at the green-berry stage, and 1262, 2638, and 2294 DEGs were identified at the veraison stage (Fig. 4b, Table S3). In each comparison, 57.9–68.9% of the DEGs were upregulated in the hetero-grafts. In general, we found three major trends. First, a large number of DEGs were identified in the three hetero-grafted samples compared with the auto-grafted sample, suggesting that hetero-grafting had a significant impact on the transcriptome profile. Second, more DEGs were found at the green-berry stage than at the veraison stage, indicating that there were greater differences in the transcriptome early in the berry developmental process. Third, the majority of DEGs were present in at least two of the three hetero-grafts (Fig. 4b), suggesting that the three rootstocks may have some similar effects on berry skin.

To highlight the potential biological functions of the DEGs, they were subjected to gene ontology (GO) enrichment analysis. Biological process enrichment analysis showed that 263 GO terms were highly over-represented in comparisons between the rootstocks and the auto-grafted control (Table S4). These GO terms were mainly related to response to stimulus (GO:0050896), small molecule metabolic process (GO:0044281), signal transduction (GO:0007165), and secondary metabolic process (GO:0019748) (Fig. 4c). We next considered the 2774 DEGs that were common to all the three hetero-grafts compared to the auto-grafted control at two developmental stages. They were predominantly upregulated (62.3%) and mainly involved in flavonoid metabolic process (GO:0009812) and anthocyanin-containing compound metabolic process (GO:0046283) (Table S5).

MapMan analysis was performed to evaluate the metabolic pathways of these DEGs. The results showed that the DEGs were mainly involved in phytohormone action, cell wall organization, solute transport, transcriptional regulation, and secondary metabolism (Table S3). Transcription factors were the main genes that participated in transcriptional regulation, and the most highly represented families were MYB, WRKY, AP2/ERF, and NAC. Among the secondary metabolism pathways, these DEGs were mainly concentrated in the flavonoid, terpene, and phenylpropanoid pathways.

To validate the expression profiles obtained from the RNA-seq data, nine genes were randomly selected for qRT-PCR analysis. The selected genes were mainly involved in key points of the flavonoid pathway. As shown in Fig. S2, the qRT-PCR expression profiles were similar to the RNA-seq results, suggesting that the expression profiles obtained from the RNA-seq data were reliable for subsequent analysis.

### Rootstocks, especially 5BB, increase transcript levels of stilbene synthesis genes

**Figure 5:**
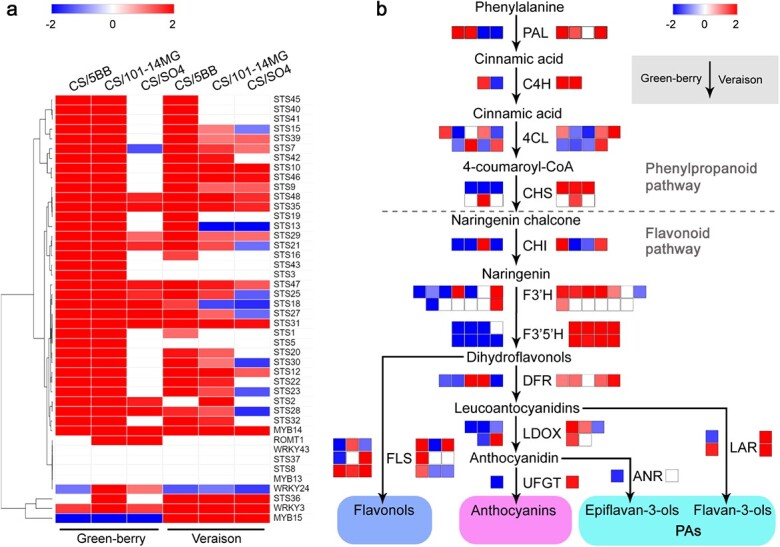
Transcriptional changes in genes related to stilbene and flavonoid synthesis pathways. **a** Expression heatmap of stilbene synthesis pathway genes at two developmental stages. Different colors represent the upregulation (red) or downregulation (blue) of the gene expression fold-change as indicated in the color index. **b** Expression patterns of phenylpropanoid- and flavonoid-related genes affected by the rootstock genotype 5BB at two developmental stages. Each colored box represents one gene, and the color of the box represents the transcriptional fold-change (blue indicates downregulation and red indicates upregulation) of this gene between CS/5BB (or the other two hetero-grafts) and CS/CS. The boxes on the left of the arrow represent transcriptional changes in related genes at the green-berry stage, and the boxes on the right of the arrow represent transcriptional changes in related genes at the veraison stage. PAL, phenylalanine ammonia lyase; C4H, cinnamic acid 4-hydroxylase; 4CL, 4-coumarate:CoA ligase; CHS, chalcone synthase; CHI, chalcone isomerase; F3′H, flavonoid 3′-hydroxylase; F3′5′H, flavonoid 3′5′-hydroxylase; DFR, dihydroflavonol-4-reductase; LDOX, leucoanthocyanidin dioxygenase; FLS, flavonol synthase; UFGT, UDP-glucose:flavonoid-3-O-glucosyltransferase; ANR, anthocyanidin reductase; LAR, leucoanthocyanidin reductase.

Stilbenes constitute a non-flavonoid class of phenolic compounds that not only act as a powerful defense system in response to biotic stresses but also have nutraceutical and pharmacological properties [32, 33]. Stilbene synthesis usually occurs in the grape berry skin [34]. In the current study, we observed enriched stilbene-related DEGs in the comparisons between hetero-grafted grapevines and the auto-grafted control (Fig. 5a, Table S6). In particular, these DEGs were almost exclusively upregulated. Stilbene synthase (STS) is the key enzyme that contributes to the biosynthesis of stilbene, and 32 of the total 37 STS genes were upregulated in the hetero-grafts (Fig. 5a). At the green-berry stage, both 5BB and 101-14MG rootstocks induced high expression of these genes, with 27 STS genes showing up to 76-fold upregulation. By contrast, only 3 STS genes were upregulated in CS/SO4 at this stage. At the veraison stage, there were still 23 upregulated STS genes in CS/5BB, compared with only 7 and 2 upregulated STS genes in CS/101-14MG and CS/SO4 (Fig. 5a). These results suggested that STS genes were differentially expressed depending on the rootstock genotype and berry developmental stage. In addition, several known transcriptional regulators of the stilbene biosynthetic pathway were also investigated. The R2R3-MYB gene *VvMYB14* (VIT_07s0005g03340) exhibited increased expression in the hetero-grafts at both stages (Fig. 5a). The *VvMYB15* gene (VIT_05s0049g01020) was strongly downregulated in the three hetero-grafts at the green-berry stage but showed increased expression at the veraison stage, indicating that it probably had an initial activation at the veraison stage. Besides the MYB genes, several WRKY transcription factors such as WRKY3 and WRKY 24 have been reported as potential STS regulators [35]. We found that the expression levels of *VvWRKY3* (VIT_01s0010g03930) and *VvWRKY24* (VIT_08s0058g00690) were significantly upregulated only in CS/101-14MG at the green-berry stage (Fig. 5a). At the veraison stage, all three rootstocks induced high expression of *VvWRKY3*. The high transcriptional levels of STS and regulators reported above were observed in the three hetero-grafts, particularly CS/5BB, suggesting a putative increase in stilbene metabolite synthesis and accumulation in the berry skins associated with scion–rootstock interactions. A previous study investigated the stilbene concentrations of 78 *V. vinifera* varieties for 3 years and found significant differences among the genotypes [34]. The current study found that the rootstock genotype 5BB had the greatest effect on the transcription of stilbene synthesis genes, suggesting induced stilbene content that was beneficial to the accumulation of phenolic compounds in the berry skin.

### Rootstocks promote transcription of genes related to the flavonoid biosynthesis pathway

The flavonoid pathway leads to the synthesis of various phenolic compounds, including flavonols, anthocyanins, and PAs [29]. Therefore, we detected the expression of early phenylpropanoid-, anthocyanin-, and PA-related genes between hetero-grafts and the auto-grafted control to corroborate the metabolic data (Fig. 5b, Table S6). Cinnamic acid 4-hydroxylase (C4H), phenylalanine ammonia lyase (PAL), and 4-coumarate:CoA ligase (4CL) are key enzymes that catalyze the first three steps of the phenylpropanoid pathway. In the current study, the majority of these genes were differentially expressed in the comparisons between hetero-grafted grapevines and the auto-grafted control (Fig. 5b). At the green-berry stage, two PAL genes, one C4H gene, and four 4CL genes were highly upregulated in the hetero-grafts, particularly CS/101-14MG. Three PAL genes, two C4H genes, and one 4CL gene were significantly induced in the three hetero-grafts at the veraison stage. It was noteworthy that the expression levels of *VvPAL2.2* (VIT_13s0019g04460), *VvC4H1* (VIT_06s0004g08150), and *Vv4CL2.2* (VIT_11s0052g01090) were activated in all three hetero-grafts at the veraison stage. The high expression levels of these genes suggested higher accumulation of the substrate 4-coumaryl-CoA, which would ultimately affect downstream pathways.

Phenolic compounds such as proanthocyanidin and anthocyanin have a common synthetic pathway, and their pathways branch after coumaroyl-CoA. Chalcone synthase (CHS) and chalcone isomerase (CHI) mediate the reaction from coumaroyl-CoA to naringenin. At the green-berry stage, three CHS genes (CHS1) and three CHI genes were downregulated, whereas only one CHI gene, *VvCHI3* (VIT_19s0014g00100), was highly activated in the hetero-grafts (Fig. 5b, Table S6). By contrast, the majority of CHS and CHI genes were significantly induced in the three hetero-grafts at the veraison stage. Flavonoid 3′5′-hydroxylase (F3′5′H) and flavonoid 3′-hydroxylase (F3′H) participate in catalyzing the hydroxylation of the B-ring of naringenin and dihydrokaempferol. In the current study, transcriptional profiles of F3′5H genes differed markedly between the two developmental stages. In contrast to the auto-grafted control, the three hetero-grafts showed lower expression of seven F3′5H genes at the green-berry stage, but their expression levels increased significantly at the veraison stage. F3′5′H activity usually prevails over that of F3′H, and we indeed observed that only a few F3′H genes were differentially expressed between hetero-grafted grapevines and the auto-grafted control. Three upregulated and three downregulated F3′H genes were identified in the hetero-grafts at the green-berry stage, and only two upregulated F3′H genes were found at the veraison stage.

Flavonol synthase (FLS) is a key enzyme on the flavonol branch. We observed that *VvFLS6* (VIT_02s0012g00400) was highly expressed in the hetero-grafts at both developmental stages, whereas *VvFLS10* (VIT_13s0047g00210) and *VvFLS* (VIT_18s0001g03470) were upregulated only at the veraison stage (Fig. 5b, Table S6). Dihydroflavonol-4-reductase (DFR), leucoanthocyanidin dioxygenase (LDOX), and UDP-glucose:flavonoid-3-O-glucosyltransferase (UFGT) are involved in the biosynthesis of anthocyanins. Transcripts of three DFR genes (VIT_18s0001g12800, VIT_13s0064g00290, and VIT_03s0038g04220) increased to higher levels in the hetero-grafts than in the auto-grafted control. Four downregulated and one upregulated LDOX gene were identified in the hetero-grafts at the green-berry stage, and three upregulated LDOX genes were found at the veraison stage. In addition, transcripts of *VvUFGT1* (VIT_16s0039g02230) were very low in the three hetero-grafts at the green-berry stage, but they increased markedly at the veraison stage. Leucoanthocyanidin reductase (LAR) and anthocyanidin reductase (ANR) provide two separate pathways for synthesizing the starting units of PA polymers. *VvLAR1* (VIT_01s0011g02960) showed lower transcript levels in the three hetero-grafts at the green-berry stage but was significantly upregulated at the veraison stage. Transcripts of *VvLAR2* (VIT_17s0000g04150) were highly increased in all three hetero-grafts compared with the auto-grafted control at both stages. In addition, we observed that *VvANR* (VIT_02s0025g01260) showed higher transcript levels in the hetero-grafts at the green-berry stage. Taken together, the above results indicate that the rootstocks promoted flavonoid synthesis at both developmental stages.

### Rootstocks activate transcription factors

Functional enrichment analysis of the DEGs between hetero-grafted grapevines and the auto-grafted control revealed a large number of transcription factors (TFs), which were mainly involved in gene regulation, modification, and signaling [36]. In the current study, a total of 396 TFs, accounting for 42% of total TFs, were differentially expressed during berry development, suggesting that rootstocks may utilize numerous TFs to regulate downstream genes. In general, the number of upregulated TFs was greater at both stages. Comparison between the two developmental stages showed that more DEGs encoding TFs were observed at the green-berry stage, indicating that TFs were more active at this stage. In addition, among the three hetero-grafted combinations, CS/101-14MG had more differentially expressed TFs at the green-berry stage, and CS/101-14MG and CS/SO4 had more differentially expressed TFs at the veraison stage, suggesting that the rootstock genotype 101-14MG promoted the activation of TFs during berry development. Further analysis showed that these DEGs could be divided into 29 TF families, and the most highly represented families were the MYB, AP2/ERF, C2C2, and WRKY TFs (Fig. S3). MYB TFs such as MYB14 and MYB15 have been reported to participate in the feedback regulation of stilbene biosynthesis. In the current study, they were differentially expressed in the hetero-grafts during berry development. Although the biological functions of the large number of remaining differentially expressed MYB TFs were unclear, we speculated that they might participate in some important pathways such as the biosynthesis and regulation of phenolic compound pathways.

## Discussion

**Figure 6:**
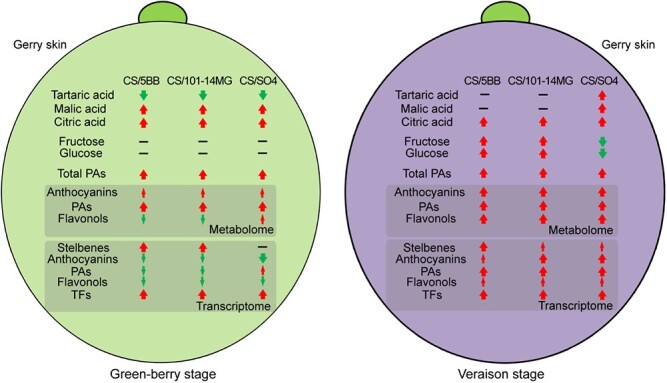
Response of grape berry skins at different developmental stages affected by different rootstocks based on a combination of physiological, metabolomic, and transcriptomic data. The red or green arrow represents an increase or decrease, and the thickness of the arrow indicates the degree of influence.

Grafting is a widely used strategy in viticulture that affects grape development as well as fruit and wine quality [25]. Phenolic compounds are primarily located in the grape berry skin and not only protect against biotic and abiotic stresses but also contribute to the taste and astringency of wine [37]. Some studies have revealed that grafting has an influence on the chemical composition and transcriptome of the berry skin at maturity [38]. However, our understanding of the effects of rootstocks on berry skin during berry development remains limited. In the current study, we characterized the physiological, metabolic, and transcriptomic differences caused by three different scion–rootstock combinations by comparing them with an auto-grafted control. Our results showed that hetero-grafting produced great changes in the berry skin (Fig. 6).

At the green-berry stage, cell division is rapid and berry size undergoes a sigmoidal increase [27]. Grape organic acids are synthesized and reach maximal concentrations at this stage. Consistent with this scenario, we observed that the concentrations of organic acids in the berry were much higher at this stage than at the veraison stage. Furthermore, all three hetero-grafts contained more malic acid and citric acid than the auto-grafted control, indicating that rootstocks could promote the accumulation of organic acids. Conversely, sugar content was relatively low at this stage, probably because sugar accumulation usually begins prior to veraison [24]. Total PA content was much higher in the hetero-grafts, especially 5BB. We focused on the effects of rootstocks on phenolic compounds and performed metabolomics analysis. The results showed that 105 flavonoid compounds were differentially accumulated at the green-berry stage, and they were mainly composed of anthocyanins, PAs, and flavonols, consistent with some major trends described previously [4, 39]. All three rootstocks showed significantly increased abundance of many PA compounds. However, only five anthocyanin compounds were present in higher amounts in the hetero-grafts. We also observed that only SO4 increased the accumulation of flavonol compounds. Further transcriptomic analysis identified several thousand DEGs between hetero-grafts and the auto-grafted control, and many DEGs were involved in phenolic compound pathways. The rootstock genotypes 5BB and 101-14MG increased the transcript levels of stilbene synthesis genes, whereas SO4 did not. Notably, the rootstocks slightly decreased the expression of genes involved in the biosynthesis of anthocyanins and PAs. Similarly, another rootstock genotype, 1103 Paulsen, was also found to have a strong influence on ripening processes related to secondary metabolite accumulation in grape berries [38]. As important regulators, many TF genes such as MYB and WRKY genes exhibited higher transcript levels in hetero-grafted plants in this study. Some of these TFs were key regulators of the phenylpropanoid pathway [40, 41]. More DEGs encoding TFs were observed at the green-berry stage, and 101-14MG had more differentially expressed TFs, reinforcing the hypothesis that this rootstock has a strong modulation effect.

At the veraison stage, there is no increase in berry size, but sugar content begins to accumulate [27]. According to the physiological analysis, both fructose and glucose contents increased markedly compared to those at the green-berry stage [42]. Of the three hetero-grafts, CS/5BB and CS/101-14MG exhibited much higher sugar contents than CS/SO4 and the auto-grafted control, indicating that these two rootstocks promoted the accumulation of fructose and glucose. Although organic acid content decreased at this stage, we still found that the rootstocks, especially SO4, increased the accumulation of organic acids in the berry skin. Notably, the three rootstocks not only promoted the accumulation of total PA but also increased the concentrations of multiple PA components. Among the three hetero-grafts, we observed that CS/5BB had the highest concentrations of seven PA components, suggesting that 5BB may significantly promote the accumulation of PAs in berry skin. Comparative metabolomics analysis showed that all three rootstocks significantly promoted the accumulation of flavonols and PAs. Consistent with this finding, the rootstock genotype SO4 has been reported to promote a strong increase in PAs and anthocyanins in berry skin [43]. Further transcriptomic analysis showed that the rootstocks induced the transcript levels of genes involved in the synthesis of stilbenes, anthocyanins, flavonols, and PAs. In addition, a large number of genes encoding TFs were upregulated in the hetero-grafts. Taken together, these data suggest that the three rootstocks had an influence on phenolic compound pathways at the veraison stage.

In conclusion, our results based on a combination of physiological, metabolomic, and transcriptomic approaches support the hypothesis that grafting with rootstocks has beneficial effects on grape berry skin and can promote the accumulation of phenolic compounds, including stilbenes, anthocyanins, PAs, and flavonols. We speculate that there are two possible reasons for these findings. First, rootstocks affected the transcript levels of genes in the phenolic pathway. The expression levels of many genes related to the synthesis of stilbenes and flavonoids (anthocyanins, PAs, and flavonols) were increased in the hetero-grafts compared with the auto-grafted control. Second, rootstocks affected the transcript levels of many TFs (such as MYB and WRKY TFs) that participate in transcriptional or post-transcriptional regulation of genes in the phenolic compound biosynthesis pathway. These results can provide new insights for improving the practical value of grafting by enhancing the accumulation of nutritious phenolic components in grapes.

## Materials and methods

### Plant materials and sample collection

The experiments were conducted in the Anningqu vineyard of Urumqi, Xinjiang. This area is 480 m above sea level, with a temperate arid and semi-arid continental climate and a long sunshine period. The annual average temperature is 7.13°C. The grape scion used in the current study was *V. vinifera* cv. Cabernet Sauvignon (CS), and the rootstock genotypes were 5BB (*Vitis berlandieri* × *Vitis riparia*), 101-14MG (*V. riparia* × *Vitis rupestris*), and SO4 (*V. berlandieri* × *V. riparia*). In the spring of 2020, when the new shoots of CS and the rootstocks were in the semi-lignified stage, CS was hetero-grafted with each of the three rootstocks. CS was also auto-grafted with itself to serve as the control. The soil, frame, shaping, and water and fertilizer management were consistent among treatments.

Grape berry samples from each scion–rootstock combination were collected at the green-berry stage (45 days after flowering) and the veraison stage (75 days after flowering). At each sampling date, three independent biological replicates were randomly obtained, and each consisted of 90 berries from a different group of 6 vines. Thirty berries were frozen immediately and stored for subsequent detection of sugar and organic acid contents, and another 30 peeled berries were frozen and stored for detection of total PAs and PA components. For the remaining 30 berries, skin tissues were separated from the pulp, frozen immediately in liquid nitrogen, and stored at −80°C for metabolomic and transcriptomic analyses.

### Measurement of sugars and organic acids and metabolomics analysis

The sugar content of grape berries was measured as described in “Determination of fructose, glucose, sucrose, maltose, lactose in foods-High-performance liquid chromatography (GB/T 22221-2008)”. The organic acid content of grape berries was measured as described in “Determination of organic acids in plant-Liquid chromatography-tandem mass spectrometry (GB/T 40179-2021)”. The total PA concentration of the berry skin was measured as described in “Determination of tannin content in fruit, vegetable and derived product-Spectrophotometry method (NY/T 1600-2008)”. The grape berry skin samples were freeze-dried and ground into powder, followed by qualitative and quantitative analysis of flavonoid compounds using the UPLC-ESI-MS/MS system as described by Sun et al. [36]. Statistical analysis was performed with SPSS 22.0 statistical software. Differences between two groups were analyzed by one-way ANOVA followed by Tukey’s multiple comparison test.

### Metabolomic profile detection and analysis

In brief, the samples were immersed in liquid nitrogen, ground to a fine powder, suspended in 70% methanol, vortexed, and centrifuged at 20 000 rpm for 20 min at 4°C. The supernatant was filtered through a 0.22-μm nylon syringe filter and analyzed with a UPLC-QTRAP-MS system (SHIMADZU Nexera X2/Applied Biosystems 4500 QTRAP). Each sample was analyzed three times. The analysis was performed on an Agilent SB-C18 column (2.1 × 100 mm, 1.8 μm). Mobile phase A was water with 0.1% formic acid, and mobile phase B was 0.1% formic acid in acetonitrile solution. The gradient elution procedure was set as follows: 0–9 min, 0–95% B; 9–10 min, 95% B; 10–11 min, 95–5% B; 11–14 min, 5% B. The flow rate was 0.35 mL/min, and the column temperatures were held constant at 40°C. The injection volume was 4 μL. The product ion scan was acquired using multiple reaction monitoring (MRM) mode. The raw data detected by LC-MS-MS were loaded on the metabolites database (MWDB METWARE database) for the identification of metabolites. The processed data were then imported into SIMCA-P14.1 software (Umetrics, Umea, Sweden) for principal component analysis (PCA) and orthogonal PLS-DA analysis (OPLS-DA). The quantitative analysis of metabolites was performed using Analyst 1.6.3 software. The metabolites were also analyzed using the KEGG database (http://www.kegg.jp) to resolve their metabolic pathways.

### RNA isolation and whole-transcriptome sequencing

Total RNA was extracted from berry skin tissue samples from each scion–rootstock combination using the RNAsimple Total RNA kit (Tiangen, China) based on the manufacturer’s protocol, then treated with DNase I to remove DNA contamination. The obtained RNA was detected using gel electrophoresis and quantified with a NanoDrop spectrophotometer. RNA integrity was verified using the Agilent 2100 Bioanalyzer. For each sample, a cDNA library was constructed with the TruSeq RNA Sample Preparation Kit V2 (Illumina, USA) following the manufacturer’s protocol. The prepared libraries were sequenced using the Illumina sequencing platform.

### Transcriptomic analyses

The *V. vinifera* reference genome was obtained from NCBI. The low-quality RNA-seq reads were discarded, and the adaptors were clipped. High-quality reads were then mapped to the *V. vinifera* genome using HISAT2 software with default parameters [44]. Uniquely mapped reads were used for subsequent analysis. The expression value of each grape gene was calculated by normalizing to the RPKM value (reads per kilobase of transcript per million mapped reads). The expression values of each sample were converted to z-scores and subjected to PCA analysis. Differentially expressed genes (DEGs) between two samples were identified using DESeq2 software [45] with a significance threshold of *p*-value <0.05 and fold-change >2 or < 0.5. Gene ontology (GO) enrichment analysis of DEGs was performed with Blast2GO software [46]. The metabolic pathways of the DEGs were analyzed using the MapMan ontology tool [47].

### Quantitative real-time PCR analysis

To confirm the accuracy of the transcriptome profiling, the expression of nine randomly selected genes was evaluated by qRT-PCR analysis. Total RNA was extracted from berry skins of each hetero-graft at two developmental stages. Actin was used as the endogenous control. Real-time monitoring of PCR was carried out with the ABI 7500 Real Time System (Applied Biosystems) using the following parameters: 95°C for 5 min, 95°C for 10 s, and 60°C for 34 s (40 cycles) to calculate cycle threshold values, followed by a dissociation program of 95°C for 15 s, 60°C for 1 min, and 95°C for 15 s to obtain melting curves. Data analysis was performed with the 2^−ΔΔCt^ method.

## Supplementary Material

Web_Material_uhac055

## Data Availability

The raw RNA-seq reads have been deposited at the National Center for Biotechnology Information (NCBI) and can be accessed in the Sequence Read Archive (SRA) database (http://www.ncbi.nlm.nih.gov/sra) under accession number PRJNA777448.
