# Supplementary figures and images for: Grafting with rootstocks promotes phenolic compound accumulation in grape berry skin during development based on integrative multi-omics analysis
Source: Hortic Res. 2022 Mar 14;9:uhac055. doi: 10.1093/hr/uhac055 (PMC9154076; doi:10.1093/hr/uhac055)

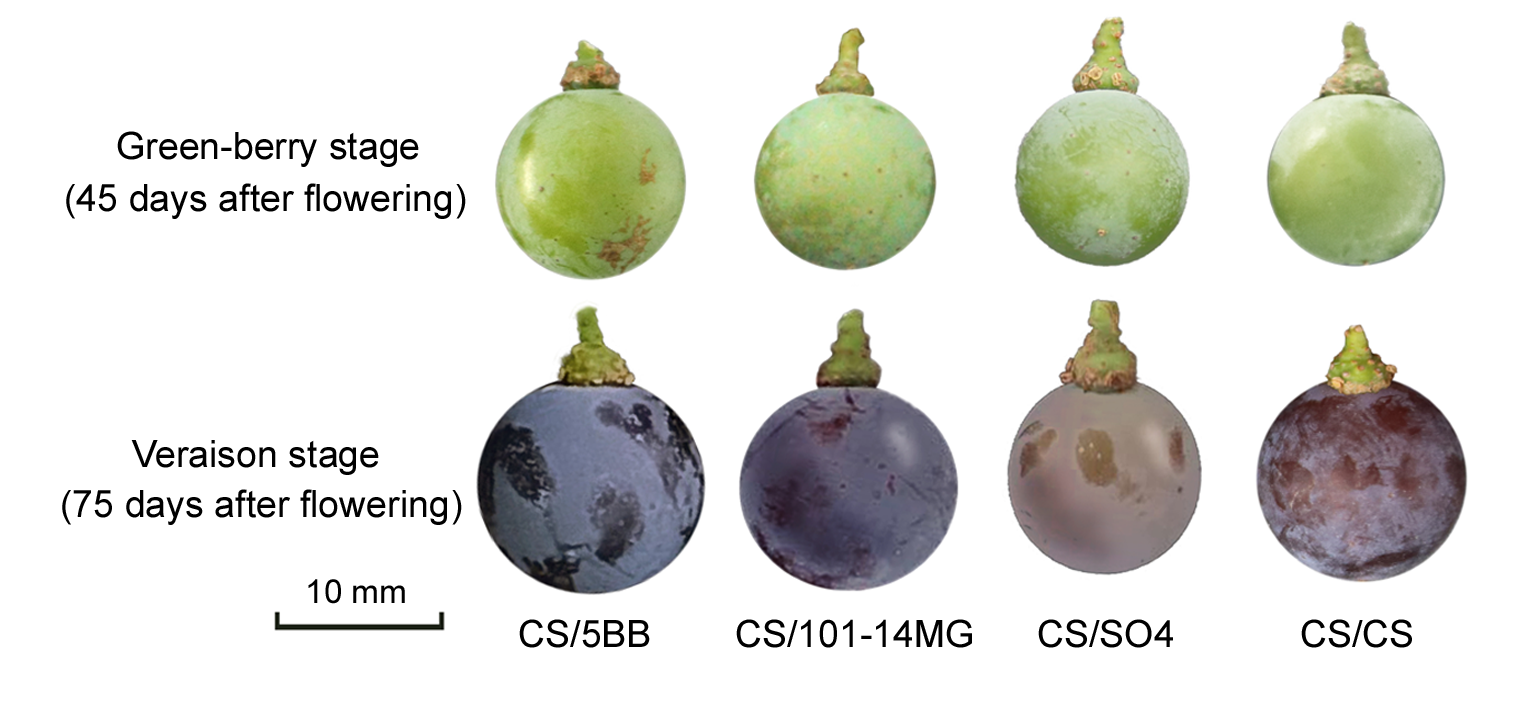

Supplement: Web_Material_uhac055 [file web_material_uhac055.zip › Fig S1.tif]

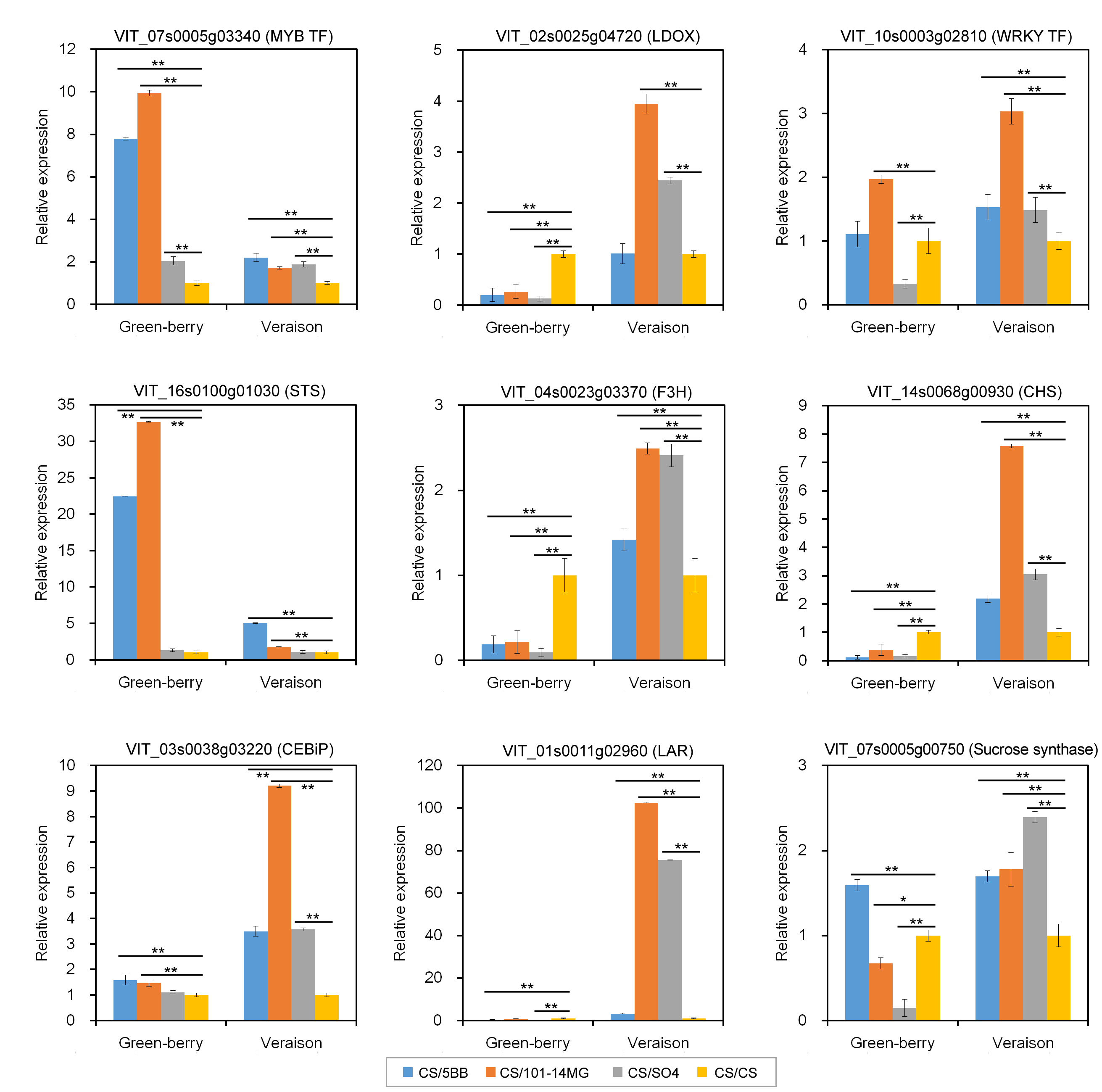

Supplement: Web_Material_uhac055 [file web_material_uhac055.zip › Fig S2.tif]

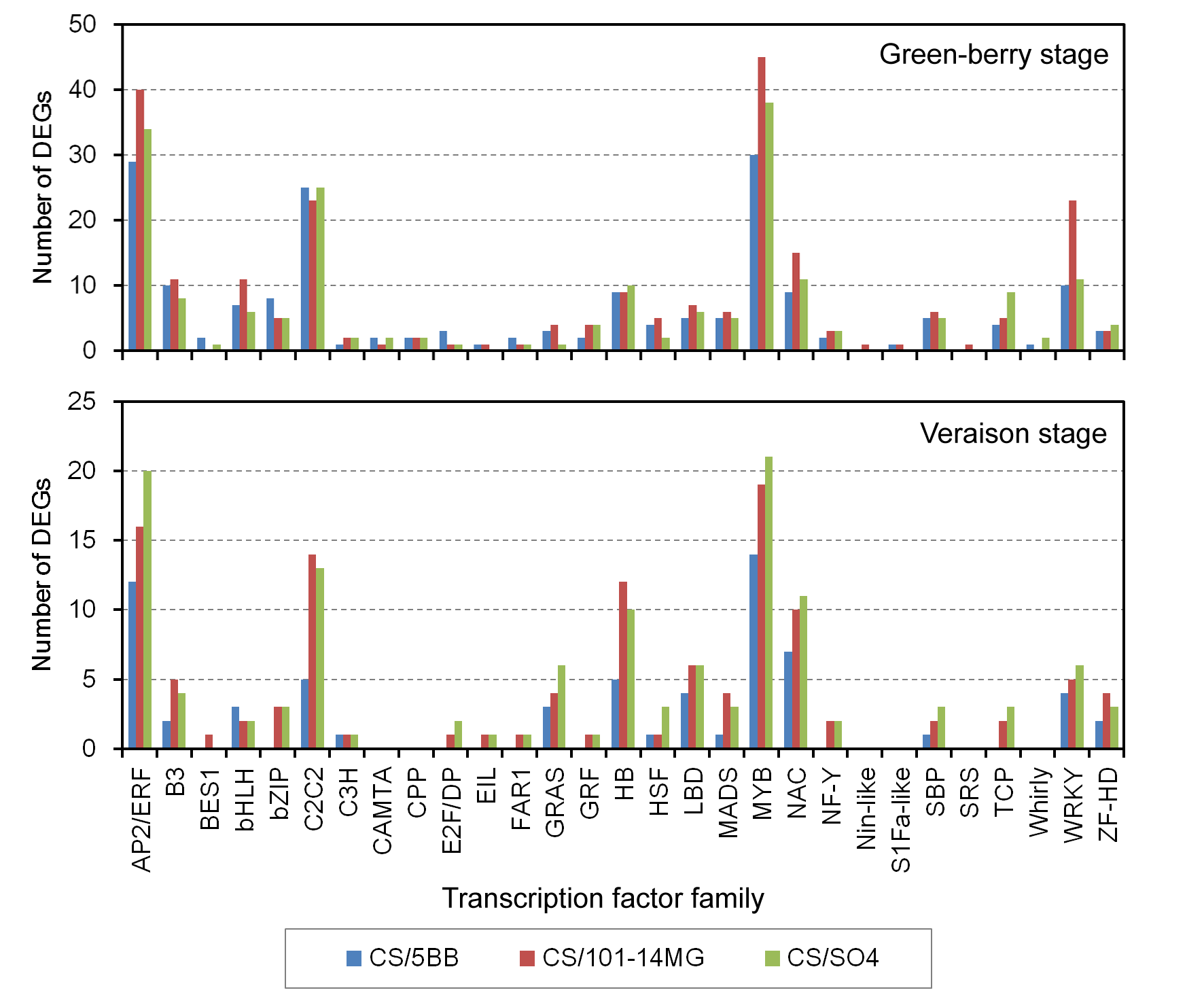

Supplement: Web_Material_uhac055 [file web_material_uhac055.zip › Fig S3.tif]
